# "The Hospital" Nursing Mirror

**Published:** 1901-05-18

**Authors:** 


"The Hospital" Nursing Mirror.

Being the Nursing Section of "The Hospital."

[Contributions for this Section of "The Hospital" should be addressed to the Editor, "The Hospital" Nursing Mirror, 28 & 29 Southampton Street Strand, London, W.C.]

### Notes on News from the Nursing World.

### JAPANESE ARMY NURSES.

Some exceedingly interesting particulars respecting the nurses trained by the Japan Red Cross Society for the purpose of employing them under the direction of the army authorities in tending the wounded and sick in time of war or on the occurrence of a natural catastrophe, are supplied to us by the manager. In sending them to us from Tokio he says that the nurses are called to actual service only when extraordinary events make it necessary to undertake the work of relief, and he adds that the hospital ships having been ordered to suspend their charitable functions on the sea while the Peiho is ice-bound, the sanitary corps on board ship were temporarily released from their duties in November. It will be observed that the course of training is for three years, and that the instruction given to probationers runs very much on English lines.

### THE NURSES OF PRINCE ALFRED HOSPITAL, SYDNEY.

At the annual meeting of the Governors of and subscribers to the Prince Alfred Hospital, Sydney, last month, there were several interesting features. The matron's report showed that steady progress had been made by the pupil nurses during the year. In December forty-one probationers and pupil nurses entered for the theoretical examinations. Of this number two succeeded in gaining first-class honours, and sixteen second-class honours. The number of candidates for training as nurses still continued far in excess of requirements. During the year no fewer than 223 applied to have their names enrolled for vacancies on the nursing staff. In October, 1899, the new rules for nurses came into force, one of the most important changes being the extension of the period of training from three to four years. According to the new curriculum, it was proposed to give a post graduate course during the fourth year, the subjects being midwifery, dispensing, housekeeping, and probably another, not yet selected. In moving the adoption of the general report, Professor Anderson mentioned that Sister Fletcher was offered the management of the branch of the Yeomanry Hospital, which he considered was a great distinction. Dr. Graham, Mayor of Sydney, said he was glad that a huge union had been formed of all the nurses of the colony for their benefit and protection. Finally, the prizes were presented to the nurses, Miss S. B. M'Gahey, the matron, calling out the names. There were 41 probationers and pupil nurses examined. Of these 14 were probationers, 16 were second year nurses, 11 were third year nurses. The following obtained prizes:—Third year: First prize, Nurse Scobie; second prize, Nurses Morgan and Morse (equal). Second year: First prize, Nurse Vernon; second prize, Nurse Davis. Special prizes in second year: For practical nursing—First prize, Nurses Miller and Moors (equal). First year: First prize, Probationer West; second prize, Probationer Barton. Special prizes in first year: For invalid cookery—First prize, Probationer Watkins; second prize, Nurses Bowden and Walker (equal).

### THE COLONIAL NURSING ASSOCIATION.

The annual meeting of the Colonial Nursing Association will be held at Londonderry House next Wednesday afternoon at 3.30. Earl Grey will preside, and among the speakers will be Lord Onslow, Lady Balfour of Burleigh, Colonel Sir James Wilcocks, and Mr. Winston Churchill, M.P. Lord Grey will be able to tell the meeting something about the progress of the nursing movement in Rhodesia.

### NURSES WANTED IN WESTERN AUSTRALIA.

Some interesting particulars are given in another column about nursing in Western Australia. Our correspondent is able to confirm, from her own experience, our statement, made some time ago, that there is an opening for good nurses in the colony. Though emphasising the drawbacks, she does justice to the advantages offered, and intimates that "sensible, hard-working nurses who could bear disappointment and defeat, and yet go on trying to raise the tone of the work to a higher standard than exists at present," would be appreciated. We desire to emphasise this point, and to warn those who are not able to accept the conditions of nursing in existing circumstances, while cherishing the hope of improving them, not to take risks which they might bitterly regret.

### FORMATION OF A NEW ORGANISATION IN NEW YORK.

Last month the Convention of graduate nurses in New York met for the purpose of resolving itself into an association to be known as the "New York State Nurses' Association." The object of the association is "to secure State recognition for graduate nurses, and eventually their proper registration, uniform requirements for entrance into training schools, courses of study and examinations, and any further legislation which will secure the betterment of the trained nursing profession and its ultimate well being." Fifty-five nurses were present and assented to the articles of the Association. But what are they among so many?

### NURSING AT CAPETOWN.

In contradiction to the statements made in our columns by "Delta" on the 30th of March, a nurse of wide experience who is now in Cape Colony, and who has an excellent knowledge of general nursing in South Africa, informs us that numbers of trained nurses are doing excellent work in Capetown in private nursing, "many of whom are neither young nor handsome." She continues: "Nor are there to my knowledge many pretty, half-trained nurses doing duty in our military hospitals; indeed, the great causes for complaint among colonial nurses is that they, although fully trained, have not been employed by the military authorities as they would have wished. It is true that to the regret of all interested in nursing matters nurses with scarcely any training were employed on transports. I fear that your correspondent has had an unfortunate experience, but this should not deter others from venturing where so many have succeeded. The time however has not come to start nursing homes up-country. We are beset with war and plague, and no one should attempt anything of the kind yet."

### PAPER "NURSES" AT YORK WORKHOUSE INFIRMARY.

THE York Board of Guardians have been sharply rapped over the knuckles by Mr. Bagenal, the Local Government Board Inspector, who has been investigating the administration of the workhouse. At a meeting of the Guardians he criticised the arrangements for attending on the imbeciles and sick, and said that during the course of his inspection he came across four paupers asleep in their beds. On asking the cause, he was informed that they had been on night duty in the sick wards, and he therefore concluded that the practice of employing pauper nurses by night, which was supposed to be stopped by the Nursing Act of 1897, is still carried on in York Workhouse. Mr. Bagenal insisted that the staff of nurses for patients is inadequate, and t hat in order to both improve the sick wards and to provide the nurses with proper accommodation, it is a matter of urgent necessity to erect a nurses' home on land adjoining the workhouse, which has recently been acquired by the Guardians. An effort was made by some of the Guardians to show that they were not in fault, and had effected several improvements, but Mr. Bagenal promptly replied that "they must go on improving." They had, he continued, to conform to the standard of the treatment of the sick which the public had decided should prevail, and he maintained that the first essential step was to properly house the nurses. With a suitable nurses' home he believed that the Guardians would get a better class of nurse, who would stay, instea d of leaving as thev often do now. At least, it may be presumed, the Guardians will, in future, obey the Act of 1897.

### ARE STAFF NURSES SCARCE?

The Grimsby and District Hospital recently advertised for a staff nurse, but we are informed that owing to none of the candidates being "suitable" it has been determined "to manage with an extra probationer." This suggests a strange scarcity of staff nurses, and does not tend to confirm the theory which "A Somersetshire Nurse" broaches in another column, that applications for such posts are so numerous that the stamps enclosed for reply and not returned cover the cost of advertising.

### A NURSES' BAZAAR.

THE three days' bazaar in Shoreditch Town Hall, last week, was very successful. The object was to raise £500 to clear off a debt on the Building Fund for the enlargement of the Hoxton and Haggerston District Nurses' Home in Nichol Square; and we understand that the takings at the stalls for the three days amounted to £528. It is hoped that the entertainment and ticket money will cover all expenses. The room in which the bazaar was held looked very bright and gay; the stalls were loaded with "a goodly show," and flags festooned above added to the pretty effect. The Marchioness of Londonderry opened the bazaar on Wednesday, the Countess of Selborne on Thursday, and Lady Lucy Hicks-Beach on Friday. Two of the stalls were labelled "The Nurses," and here were to be seen the nurses themselves, in uniform, and wearing badges of blue ribbon. On their stall, among other attractions, were excellent photographs of the Home, which now occupies an entire corner of the square. "The mind ought sometimes to be amused," were the words heading a page of the programme, "that it may the better return to thought and to itself." And so, besides the attractive features of the stalls, there were musical and other entertainments at intervals, including an electric battery, an exhibition of the X Rays, and a table for "Ping Pong" in the gallery. Miss Vickers' Amateur Orchestra played selections by well-known composers, and the ladies' mandoline band and the Highbury ladies' string band also performed at intervals, while other friends contributed vocal and instrumental music. There was an amusing competition for men called the "Button-sewing Competition," in which no thimbles were used; and another in hat-trimming, also for men; while the ladies had a "Sunlight Soap Washing Competition," with most tempting prizes. Altogether, there was plenty of variety, and we have no doubt that the nurses enjoyed their bazaar as much as any of the visitors. Mr. and Mrs. Snowden have kindly arranged to give a large garden party in about two months' time, when it is hoped that some of the more expensive goods still on hand may be sold.

### THE MATRONSHIP OF MACCLESFIELD INFIRMARY.

THE Macclesfield people are very much concerned lest the post of matron of the General Infirmary should not be judiciously filled. It was vacated by the resignation of Miss Noble, and it is widely desired that her successor should be a person of ripe experience who is devoted to her work, and will make the welfare of all under her care her first consideration. It is no secret that there are difficulties to encounter, and that some rearrangement of offices needful in the interests of the institution may be decided upon hereafter. But a capable woman will be quite prepared to face situations as they arise. The House Committee, in inviting applications for the appointment, have done their best by not fixing an age limit, to ensure a supply of eligible candidates.

### THE SHEFFIELD SLANDER CASE.

IT will be remembered that Miss Clarice Battye, a nurse at Fir Vale Workhouse Infirmary, Sheffield, brought an action for slander last year against Mr. Frank Shelton, the steward and caretaker of the Masonic Hall, Sheffield, who had accused her on two occasions of misconduct with a doctor at the ball of the Sheffield Society of Chemists. The defendant pleaded that the words on both occasions were spoken on a privileged occasion and without malice, but Mr. Justice Grantham held that though the first occasion was privileged, there was evidence of actual malice, and that the second occasion was not privileged. The jury accordingly found a verdict for the plaintiff and awarded her £100 damages. The defendant thought proper to appeal against this decision, and last week the case came on before the Master of the Rolls and his colleagues, with the result that the Court summarily dismissed Mr. Shelton's application. The Master of the Rolls in the course of his remarks, said that the case seemed to him to be a very clear one.

There was no pretence for saying that the accusation was true. The sole defence was that the occasion was privileged. No doubt a plea of justification was put upon the pleading, but that plea was not pressed, and it was abandoned when the case came on for trial. The first statement was made on the night of the ball, before the ball had concluded, to the president of the Chemists' Society at the ball. He (the Master of the Rolls) was not prepared to say that the occasion was not privileged. With regard to the second statement, a very different state of circumstances existed. Everyone had left the hall. On the next day the defendant made the statement complained of to the secretary of the Chemists' Society. Undoubtedly the statements were untrue. The defendant, however, contended that the second occasion was privileged. What duty had the defendant to make the statement? He could not see any duty.

Miss Battye is to be congratulated upon the complete success which has attended her efforts to vindicate her character.

### A "VICTORIA MEMORIAL HOME" AT DERBY.

A HOME OF REST for incurables, in memory of Queen Victoria, has been opened at Derby. It has been started on a very small scale. The house is pleasantly situated and contains accommodation for five patients. It has been furnished altogether through voluntary contributions, upon which it will be chiefly dependent. One of the founders of the home is Miss Atthill, lady superintendent of the district nurses of the Royal Derbyshire Nursing and Sanitary Association, from which association the necessary nurses will be supplied. It is intended for incurable patients who can only live a short time and who cannot be properly looked after in their own homes. In time it is hoped that it may grow and be a home of rest and comfort to many poor sufferers.

### REIGATE ISOLATION HOSPITAL.

IN our last issue we published a letter from a correspondent commenting upon the management of the Reigate Isolation Hospital, more especially in regard to the position in which the sanitary inspector had been placed in regard to the management of the institution. We need hardly say that in making such comment the intention was to call attention to the action of the sanitary authority, and not in any way to that of the sanitary inspector. The sanitary authority is the body responsible for the management of the hospital, and for the position occupied by each of the officials concerned, and if it is the fact, which has since been denied by another correspondent, that the sanitary inspector at Reigate has been appointed to what we consider an unsuitable position, he cannot be blamed so long as he only carries out the directions of the sanitary committee, whose servant he is. As we read the letter of "An Interested Onlooker," the intention of the writer was to criticise the arrangement under which "a sanitary inspector was appointed manager," and not in any way to animadvert upon the conduct of the inspector in the performance of the duties so laid upon him; but we must add that at a meeting of the Reigate Town Council the Mayor is reported to have said that "the matron of the hospital was entirely under the control of the sub-committee and the medical attendant."

### EAST END MOTHERS' HOME.

THE annual meeting of subscribers to the East End Mothers' Home was held last Friday, at 5 Wimpole Street, W., by permission of Dr. Owen Lankester. The chair was occupied by the Rev. Sidney Vatcher, in the absence of Major Evans-Gordon, M.P. Mr. Vatcher said he did not think the value of the home could possibly be overrated; it was the only one of the kind in the East End. Mr. Woolcombe, hon. solicitor, proposed the adoption of the report, and observed that he could endorse all that the chairman had said. The Rev. W. Muirhead, Vicar of St. Thomas's Stepney, and hon. chaplain to the home, seconded; he knew many poor women who had been treated; the home was becoming more and more useful every day. The chairman proposed that the name of Dr. May should be added to those of the consulting physicians; he had said he would gladly attend at the shortest possible notice. The Rev. F. J. Jomini proposed the re-election of the general committee and the committee of management, and this was seconded by Lady Greville, to whom, the chairman said, the foundation of the home was due. A letter had been received from the Lady Lucy Hicks-Beach regretting her inability to attend the meeting.

### WHAT IS A CO-OPERATIVE NURSING HOME?

IT seems necessary that the question "What is a Co-operative Nursing Home?" should be answered, for we are constantly receiving letters from people claiming to be carrying on co-operative homes. For instance, a fortnight ago we stated in reply to a correspondent that there is no home in Manchester managed by a committee worked on co-operative lines. We have since received a letter from the matron of the Manchester and Salford Sick Poor and Private Nursing Institution, 341 Oxford Street, Manchester, in which the writer says, "This is a co-operative nursing home, managed by a committee; we also have some nurses receiving a fixed salary." So far so good. We would point out, however, that the essential principle of a co-operative home is, not only that there should be a committee of management, but also that, after the deduction of a percentage for working expenses, each nurse should receive all she earns. Hence co-operation is incompatible with a fixed salary.

### A DISHONEST PROBATIONER.

AT Bristol Police Court the other day, a probationer was found guilty of a series of petty thefts and sent to prison for six weeks in the second division. It was pleaded in mitigation that she had taken some of the articles without felonious intent, in order to obtain a pattern from them, that her health was bad, and that she was lately married. But as several nurses in succession gave evidence as to the articles which had been purloined from them by the prisoner, including a scent-bottle with a silver top, articles of clothing, a tablecloth, a copy of Longfellow's poems, a work on physiology, and it was clear that she had been persistently guilty of stealing, the magistrates would not have been justified in overlooking her misconduct. It can only be hoped that the none too severe punishment inflicted upon her will have the salutary effect of curing her of her thieving propensities.

### SHORT ITEMS.

LORD METHUEN has forwarded a cheque for £20 to the Kimberley Hospital, together with a letter, returning thanks for the attention which he received from the doctors and nurses while he was a patient suffering from fever.—The Dowager Countess of Longford has been able to send parcels to twelve hospitals as the result of the kind response to her appeal for comforts for the nurses in South Africa. She wishes to send more, and asks for further subscriptions, however small. Each parcel contains warm underclothing and haberdashery and costs £3, including postage.—At the first annual meeting of the Hyde Sick Nursing Association it was announced that the father of the Mayor, Mr. Thomas Beeley, had presented to the association a sum equal to £40 per annum for the next five years, and that Miss Ashton, of Ford Bank, Didsbury, was giving £50 a year.—A most successful and enjoyable soirée took place recently in Mansfield in aid of the District Nursing Association. The sum of £26 12s. 10d. was handed over after paying all expenses.—A Nurses Home, costing £1,500, is to be erected at Conon as a memorial to the late Sir Kenneth Mackenzie, for some time Lord Lieutenant of the counties of Ross and Cromarty.

### Notes of Surgical Lectures to Nurses.

Delivered at the London Temperance Hospital by Dr. W. J. Collins, M.S., B.Sc., D.P.H.Lond., Fellow of London University and of the Royal College of Surgeons. Surgeon to the London Temperance and the Royal Eye Hospitals.

### ARM—BLOOD—THORAX.

Just as the pectoralis muscle it said to form the front, or anterior, wall of the axilla, so the largest and broadest of the many muscles of the back—the latissimus dorsi—forms its hinder, or posterior, wall. The action of this muscle is to move the arm backwards and downwards, at the same time rotating the hand inwards, as in swimming. In the conical axilla, enveloped in fat, are the axillary artery and vein and their branches; also a plait, or plexus, of nerves destined to supply the brachial muscles with motor power, and the skin of the arm and hand with sensation. Packed away in the hollow and scarcely felt in health, but easily defined against the upper ribs when enlarged by cancer or blood-poisoning, are the axillary lymphatic glands.

The inner border of the biceps muscle affords a ready guide to the main artery—the brachial—of the arm; a line drawn from the deepest part, or apex, of the axilla along the inner border of the biceps to the middle of the bend of the elbow, marks its course to its termination by division into the radial (pulse-artery) and the ulnar which end in two arches in the palm. It is easy to stop the pulse at the wrist by compressing the brachial artery against the humerus, since arteries carry blood from the heart to the extremities, while veins return the blood to the heart after it has coursed through the microscopic capillaries. It was from a surface vein at the bend of the elbow lying just over the brachial artery that bleeding or venesection was performed when that operation was fashionable; preparatory to the operation the veins were distended by a ligature being placed around the upper arm so as to impede the return of blood. The same spot is nowadays used for the operation of transfusion or the injection of blood (defibrinated) or warm salt and water to overcome collapse (syncope) after profuse hæmorrhage. The bony prominences of the elbow should be studied, the two condyles of the humerus, the olecranon (with the ulnar nerve on its inner side) and the rotating head of the radius, as fractures here are not uncommon. Immediately above the inner condyle the first gland encountered in passing upwards from the fingers stands sentinel, and gives alarm in poisoned (septic) wounds of the hand. Many tendons surround the wrist joint; they run in sheaths which at times inflame, causing troublesome stiffness of fingers, and small "ganglia" are common in connection with them. The ulnar artery lies over the tendons in the palm as they go to the fingers, forming the superficial arch and the radial passes under them forming the deep arch; from these arches the fingers are supplied with blood. Wounds of the palm, in consequence of this rich blood supply are apt to bleed freely. The nature and arrest of "hæmorrhage" differ according to its source, viz., arterial, venous, capillary.

The heart pumps blood into the arteries quicker than it can escape through the capillaries into the veins; the walls of arteries are thicker than those of veins to resist this blood pressure. Besides an outer tough fibrous coat, no section arteries are seen to have an inner elastic coat to permit changes in calibre and pressure, and between these is a middle muscular coat which regulates the amount of blood flowing through an artery to a limb, organ, or other part. Veins also have a threefold wall, but thinner, especially in the muscular layer, than the arteries; moreover, they are provided with valves which prevent regurgitation of blood in health, but are apt to cause varicosities when the walls of the veins degenerate. The interior of arteries and veins is rendered smooth by a delicate lining membrane composed of flat tessellated cells about 1-5000th of an inch wide; the capillaries are composed of little else beyond this pavement of "endothelial" cells. If capillaries are looked at under a microscope, in the translucent web of a living frog's foot the blood in them is seen (1) to consist largely of solid particles (corpuscles), and (2) to be in motion (circulation).

More than one-third and less than one half of blood is corpuscles, the rest is plasma (liquor sanguinis); there are about 5,000,000 corpuscles in a drop of blood as big as a pin's head. Corpuscles are of two kinds:—1. Red, non-nucleated, circular, biconcave discs, 1-3600 inch in diameter. 2. White, nucleated, amœboid, mobile leucocytes, 1-2500 inch in diameter. There are 500 red to one white.

One-thirteenth of the weight of the body is blood: its function is to purvey oxygen and nourishment to, and effete products from, the myriads of cells that compose the body—to this end it circulates. Live blood is a viscid red fluid of sp. gr. 1,055, and alkaline; when shed (and sometimes, as in thrombosis, in the body) it clots; plasma yields serum and fibrin, the latter plus corpuscles forms clot. Coagulation of the blood and contraction and retraction of the arteries are the main factors in the natural arrest of hæmorrhage. Artificial methods are based on these as (1) styptics (cautery, perchloride of iron, tannic acid, turpentine) or (2) occlusion methods (ligature, torsion, pressure).

In inflammation the relation between the capillaries and the contained blood is disturbed. Afflux, blockage, sweating out of plasma and corpuscles, causing redness, swelling, heat, and pain. This is the "series of changes which result in a tissue when it is injured, provided the injury is insufficient to at once destroy its vitality." Results: resolution, suppuration (abscess), necrosis, ulceration, sclerosis or cirrhosis. At any given moment the distribution of the blood may be roughly said to be one-quarter in heart, lungs, and large vessels; one-quarter in liver and its vessels; one-quarter in muscles, and one-quarter elsewhere. The chief factor in the circulation, the heart, and the organs of respiration, the lungs, are situate in the thorax. The bony thorax is composed of the sternum and the costal cartilages in front, the twelve ribs on either side and the twelve dorsal vertebræ at the back. The sword-like sternum consists of three pieces (manubrium, gladiolus, and xiphoid), of the ribs seven are true (articulate with sternum by costal cartilages), five are false, of which the two last are floating, or free at their anterior extremities. The first lies under the clavicle, the second is opposite the junction of the first and second pieces of the stenum, marked by a ridge (angle of Ludovici), the fourth is about the level of the nipple, the fifth follows the line of the lower border of the great pectoral muscle, the sixth, seventh, eight, and ninth are identified in the living body by visible serrations of a muscle called the serratus magnus, which moves the scapula over the upper ribs as in the action of lunging.

The base of the heart lies on the level of the third costal cartilages, extending ½ inch to the right of the sternum and 1½ inches to the left. The apex beats in the fifth intercostal space 2 inches below the nipple, and 1 inch to the inner side. The heart in the middle (mediastinum) invested with pericardium, and the lungs on either side of it covered by their pleuræ, rest below on the muscular floor of the thorax called the diaphragm. This is the chief muscle of respiration, causing by its contraction enlargement of the chest by lowering its floor; it also separates the thorax from the abdomen. The liver lies below the diaphragm under cover of the six lower right ribs; its edge can be felt just below the margin of the costal cartilages on the right side. The spleen lies opposite the ninth, tenth, and eleventh left ribs, between two imaginary lines drawn vertically from the front and back walls of the axilla.

The relation of the organs within the chest and their state in health and disease can be ascertained with considerable accuracy by *inspection* of the walls with the eye, by *palpation* with the hand, by *percussion*, and by *auscultation*.

By *inspection* are ascertained the size, shape, symmetry (flat, pigeon, barrel-shaped), local swellings, the movements of each side with respiration, and the beat of the heart either in normal place in fifth space or displaced. Measuring with tape or taking a tracing with a cyrtometer will assist such observations.

By *palpation* with the hand the beat of the heart or the thrill of the voice may be felt in health or found to be increased or diminished in disease; abnormal pulsation may be discovered and abnormal thrills and frictions may be detected.

By *percussion*, or striking the chest wall, Auenbrugger, of Vienna, in 1761, showed that further information could be obtained as to the condition of the lungs and heart. A resonant note of varying pitch (tympanitic, pulmonary, osteal), or absence of resonance (dull), indicate the presence or absence of air, fluid, solid lung, &c. There are, moreover, areas of natural dulness, as over the heart (cardiac), the liver (hepatic), and spleen (splenic).

*Auscultation* or applying the ear directly to the chest wall, or indirectly with the stethescope, as practised first by Laennec in Paris in 1816, informs of the normal and abnormal sounds of the heart and lungs. Healthy breathing yields to auscultation over the lungs a soft, vesicular, murmur; over the wind-pipes a louder, tubular, or bronchial noise; while the cardiac area is almost devoid of breath sounds. Bronchial breathing, and absence of breath sounds over the lungs, are signs of disease, showing altered quality of the contents of the chest as regards its aëration. Rattling heard over diseased lung. Then, again, the voice sounds, sounds (rhonchi, râles), or crepitation, or friction may be like the vocal thrill felt by the hand on the chest during speaking, may be intensified (bronchophony) or diminished by consolidation or rarefaction of the lung, or altered in quality, like a bleat (œgophony).

The heart sounds, likened in health to lubb-dup, may be either of them or both replaced by blowing or sawing noises (murmur, bruit).

### Nurses on board the Hospital Ships of the Japan Red Cross Society.

By the Manager of the Society.

### Their Training.

The Japan Red Cross Society trains nurses for the purpose of employing them under the direction of the army authorities in tending the wounded and sick in time of war or on the occurrence of a natural catastrophe. The candidates are required to be Japanese women, strong-bodied, fairly well educated, without household encumbrances during the training period, and of 18 to 30 years of age. They have to pass two courses of examinations. The successful candidates are admitted into dormitories of the Red Cross Society's Hospital as probationers. The course of their training runs for the period of three years divided into two terms of equal length. During the first year and a half the probationers are given instruction in scientific principles of nursing with a little training in practical work, while in the second year and a half they are made to devote themselves entirely to practical nursing. On the completion of their scientific and practical studies, the probationers are admitted to a final examination and those who have acquitted themselves well at the examination, have their names registered in the "reserve nurse" list of the Japan Red Cross Society. The reserve nurses are summoned to the Red Cross Society once every other year for the purpose of giving them review lessons. The nurses who were appointed to active service on board the hospital ships the *Hakuai-Maru* and the *Kōsai-Maru* on the occasion of the recent North China disturbances, were fully trained, as they were chosen from among the nurses then on duty at the Society's hospital.

### Their Duties.

The duties of the superintendent and other nurses who were appointed to embark on the hospital ships in connection with the recent crises in North China were as follow:

*The Superintendent Nurse.*—To superintend, under the direction of the manager, chief surgeon and surgeons, all the nurses in the ship, to inspect their work in the wards assigned to their charge, and also to oversee their general conduct.

*The Matron.*—To assign duties to the nurses in accordance with instructions of the superintendent, and also to engage in the nursing of the wounded and sick. In case a patient is found in a dangerous condition to report the fact to the superintendent, to the surgeon in charge, to the chief surgeon, and to the manager.

*Nurses.*—To engage in the nursing of the wounded and sick under the instructions of the matron. In taking rest by turns, to decrease the number of hours between, when there are dangerous cases, and also to increase or decrease it in accordance with the number of ordinary cases.

No reference is made above to the treatment of dead bodies, the transportation of patients, the disinfection of the clothes and articles in the possession of the patients, the general business in the wards and the nursing of some particular classes of patients, as these properly belong to male hands.

### Remuneration.

Nurses receive no salary while they are on the reserve list. It is only when they are on active service that they are paid. The salaries paid to the nurses who have recently served on board the hospital ships were:—

| | |
|---|---|
| The Superintendent ... | 30 yen to 32 yen monthly. |
| The Matron ... ... | 26 „ „ 28 „ „ |
| Ordinary Nurse ... | 20 „ |

In addition to these monthly salaries they received while on board ship half the sum of the respective salaries, board, and an allowance ranging from 10 to 13 yen monthly.

### Miscellaneous Matters Relating to Patients.

When a patient requests a nurse in conformity with posted notices or her verbal advices to get a precious article in his possession entrusted for safe keeping, she shall cause it to be examined in the presence of the manager and the superintendent. Upon the examination the manager shall receive it in trust, giving a signed receipt therefor to the patient. The article shall be delivered to the patient in exchange with the receipt, when he leaves the vessel.

When a patient is seriously ill and unconscious, his precious property, if any, shall be examined in the presence of a few more superiors than in the case of the above clause, and get the property duly registered so that there may not be left any doubt as to the transaction.

In case a patient desires to have a letter posted, the nurse in charge shall receive it and see to its being duly mailed through a clerk in the ship.

When a letter arrives addressed to a patient, the clerk shall deliver it to the matron, who shall give it to the nurse in charge to be delivered to the addressee.

### Nursing in Western Australia.

BY AN ENGLISH NURSE.

SOME time ago there was a good deal of discussion in our hospital in London arising out of a paragraph in the NURSING MIRROR to the effect, that there was an opening for good nurses in Western Australia, and several nurses spoke of trying to go out there. One eventually summed up enough courage to visit the Agent-General of the colony, but was merely told that if she liked to go out on her own account she probably would get plenty of work to do, but that as far as they were concerned nurses were not wanted. Then the subject was dropped, for everybody's attention was drawn to South Africa. However, unforeseen circumstances drew me to Western Australia; and therefore I will try and tell you something about the nursing.

### CHARACTERISTICS OF THE COLONY.

This colony is far behind all the other colonies in every way, so it must not be assumed that all Australian nursing is necessarily the same. Being so far behind the times, it is curious that nearly every small town has a new well-built hospital to itself. These hospitals are almost all run entirely by a "matron," assisted by a male nurse, though sometimes she has a young servant to assist also. This sounds very hard on the matron, but there is often not more than one patient in at a time. On the "fields" the nurses have to rough it, and to work hard, but they generally like it, because they have such a good time when they are off duty. They are invited to all the current gaieties, and the people on the "fields" are very kind and friendly. There are no drains out here, which trebles a nurse's work and makes a world of difference—impossible to realise until experienced. One is not surprised to find that there are no other up-to-date conveniences except electricity and telephones; the result is that nursing has to be done as best it may be without the usual appliances, or with very rough or old-fashioned articles. "Anything will do" in this country, and it is peopled with "Casual Joes" Servants are almost impossible to obtain, and no one dare order them about; they will only engage by the week and seldom stay more than a month, and their wages are enormous. "Washing" is on the same footing. Most people do it at home, but there are expensive Chinese laundries to be found. The hospitals have to wash as they can, on the premises, without many conveniences.

### INADEQUATE TRAINING.

Half of the nurses are not properly trained, some of them not at all, and the doctors do not object to this as a rule—in fact, some of them prefer untrained women to look after their cases. Any of the latter will without the slightest hesitation accept any case, from an abdominal section to a midwifery case, probably having never even heard the meaning of "antiseptic" or "L.O.S." And they pocket their three or four guineas a week with the coolest *sang-froid*. I must confess they earn it, as they often have to do some of the housework, or washing, or all of the cooking for the family. This puts properly trained nurses in a false position, because, naturally, they object to perform duties that render it impossible for them to nurse their patients properly, especially as they invariably have to do both the day and night nursing. It is uncomfortable, too, if they have to object to such matters as having meals in the kitchen or sharing their patient's bed.

### THE WONDERFUL CLIMATE.

The surgical work out here is astonishing. Every detail connected with an operation is carried on in the same don't-care, happy-go-lucky manner that seems born in the West Australian. If treated in the same way at home, the inference would be that the patient must die; but here the results are perfectly marvellous: clean wounds, happy patients, and quick convalescence. I suppose it is the climate, which is said to be the most perfect in the world. It is certainly delicious, for even if the midday sun is hot, a cool breeze generally springs up towards evening, making the air almost chilly at night.

### THE TREATMENT OF TYPHOID.

There is a tremendous lot of typhoid fever still in the Colony, but it is most prevalent on the "fields." I do not think that it develops into so severe a form of disease as it does at home; at any rate the average mortality is much less. The patients out here are allowed much more freedom while they are ill, and get much less nursing, and, perhaps, more treatment. Though treatment seems to me to be thrown away, if, for instance, "a case" drinks his medicine straight out of the bottle, guessing the quantity and the time it is due; or "some one" bathes him, by leaving a hip bath beside the bed for him to have a dip if he feels hot. There is no keeping a typhoid altogether on his back and sides—he tosses about with full permission, which, at least, prevents bed-sores. Because it is so common, it is simply called the fever, and I heard a lady remark one day: "I feel quite safe about my boy going to South Africa, because he has had fever, and they say that it is a safeguard against that terrible scourge enteric."

### GOOD NURSES WANTED.

The medical profession is well represented, and there are a few really good nurses, though a great many more are wanted—sensible, hard-working, professional nurses who could bear disappointment and defeat and yet would go on trying to raise the tone of the work to a higher standard than exists at present. But it is not the fault of the doctors, or altogether of the nurses, that the profession has not been brought up to the mark. It is the fault of the easy-going, pleasure-loving people of Western Australia who are content to sit still, letting everything drift on, while they lazily congratulate themselves that theirs is the "coming colony."

### Death in Our Ranks.

WE regret to learn that Miss May Ethel Mary Grubb died at Bournemouth, on the 7th inst., of acute pneumonia. Since 1895 she had been on the staff of the Suffolk General Hospital as probationer and staff nurse, and a correspondent says that she had endeared herself to all her fellow-workers by her sweet and gentle disposition. Only last month she left Bury St. Edmunds and obtained work on the staff of the Victoria Nursing Institute, Bournemouth, in order to be near an invalid sister. The lady superintendent, Miss Christina Forrest, and fifteen of her nurses followed her to the grave on Saturday last. Amongst the wreaths was a beautiful one composed of lilies and spirea from the staff of the Suffolk General Hospital.

### To Nurses.

**WE invite contributions from any of our readers, and shall be glad to pay for "Notes on News from the Nursing World," or for articles describing nursing experiences, or dealing with any nursing question from an original point of view. The minimum payment for contributions is 5s., but we welcome interesting contributions of a column, or a page, in length. It may be added that notices of entertainments, presentations, and deaths are not paid for, but, of course, we are always glad to receive them. All rejected manuscripts are returned in due course, and all payments for manuscripts used are made as early as possible after the beginning of each quarter.**

### The Nurses' Co-operation.

So much interest has been excited amongst the nursing profession by the crisis which has suddenly arisen in regard to the Nurses' Co-operation that we think it desirable to supplement the incomplete reference to the origin of the Association which appeared last week. That reference dealt mainly with the financial aspects of the original movement, but it is important, and a mere act of justice, to emphasise the fact that the Nurses' Co-operation originated with a nurse, Miss Mary Belcher, to whose energy and foresight the ultimate establishment of this society is undoubtedly due. Those who are interested will find the whole of the facts set forth in a series of articles entitled "A Great Movement: The Nurses' Co-operation," which appeared in THE HOSPITAL NURSING MIRROR, March 30, 1895, p. cxcii, April 6, 1895, p. vi, and April 13, 1895, p. xiii.

The facts then are as follows. In 1888 Miss Mary Belcher had written to THE HOSPITAL suggesting a combination of private nurses to protect their own interests. This led to a lengthy correspondence, which proved that some such society was needed. At the suggestion of the Editor of THE HOSPITAL a committee was formed to consider and formulate a scheme which would meet the circumstances. This committee consisted of Mr. Michelli, Mr. Ryan, Miss Belcher, Miss Napper, and Miss Honnor Morten. Miss Belcher was the originator of the scheme and a representative private nurse; Miss Napper was then the matron of the Surrey Convalescent Home; and Miss Morten, who acted on the committee as the representative of the editor of THE HOSPITAL, was the author of "The Nurse's Dictionary," "How to Become a Nurse," and other well-known books. The committee wisely took a considerable time to develop the scheme, to make it practical in every detail, and to secure the support of a sufficient number of doctors. In asking doctors of repute to support the Co-operation it was necessary to give them a guarantee that its members would be such as they could recommend. In addition to the qualification of the nurses, the rules under which they should work, respect for each individual nurse employed, and some sort of homogeneity had to be secured. In the result the committee continued to work about a year before the scheme of the Nurses' Co-operation was finally published.

When the scheme was ready, before the Nurses' Co-operaion could be set up it needed a local habitation and th e necessary funds. An estimate of these sums amounted to hundreds of pounds, and, after much consideration, the financial difficulty was met jointly by the nurses and a few of their friends, being gentlemen of means whose names were given in THE HOSPITAL last week. The editor of THE HOSPITAL felt that the public spirit displayed by the nurses like Miss Belcher and Miss Napper, who each advanced £100, rendered it necessary that they should be secured against loss of every kind in case of failure, and, in conjunction with his friend, the late Mr. W. H. Burns, he consequently guaranteed them and the other nurse contributors against loss in the event of any such contingency and placed them in a position of security by promising to repay them any money which they might lose in the venture. An office was taken at 8 New Cavendish Street, Portland Place, and on February 1st, 1891, the Co-operation was ready to supply the public with nurses. At that time it had thirty nurses on its register. Miss Honnor Morten acted as honorary secretary, and Dr. J. F. Goodhart as treasurer, whilst for superintendent the Co-operation secured Miss Hicks, who had till then been matron of the Hospital for Sick Children, Great Ormond Street, but who is perhaps best known to the public as Sister Philippa of the Egyptian campaign. The Co-operation was a business association to give work for fair pay. Speaking generally, the fees charged were two guineas a week for ordinary non-infectious cases and £2 12s. 6d. per week for special, infectious, mental, and maternity cases, half a week being charged one guinea, and nurses for the day at the rate of 10s. 6d., whilst massage was charged 5s. 6d. a visit. The terms, in short, were those of most good nursing institutions, the difference being that in the Co-operation the nurses received all they earned minus a small percentage to pay office expenses. This percentage was fixed at 7½ per cent., that is, 1s. 6d. in the pound; a modest sum which many critics in those days declared would be far too small to enable the necessary expenses to be fully met.

In the result it was found that 7½ per cent. left a good margin, and in a very short time the nurses had some hundreds of pounds in hand, and a meeting was called to determine what should be done with it. The committee at that time were mostly in favour of dividing the money among the nurses, but the editor of THE HOSPITAL attended the meeting and urged the nurses to fund the money and invest it as a reserve so as to place the Nurses' Co-operation once and for all in a position of strength and permanency. After fully discussing the question it was moved and seconded by nurse members of the Co-operation and resolved to adopt the advice of the editor of THE HOSPITAL, and in the result it appears from the accounts that upwards of £7,100 was accumulated and invested, upwards of £8,000 being apparently the sum which the present committee have sunk in the Howard de Walden Home, which is at present being conducted at a loss, and which the nurses for the most part say does not meet their requirements. The present committee of the Nurses' Co-operation have further established a charge on account of this home of an additional £1,500 which will have to be met, if it is met at all, out of the balance accruing from the unexpended portion of the 7½ per cent. commission deducted from the nurses' earnings each year, which would otherwise be invested and retained for the protection and benefit of the nurse members. This step, in conjunction with the resignation of Miss Hughes, the excellent and deservedly popular lady superintendent, and the persistent way in which the present committee have failed to take the opinion of the nurse members and to consult their wishes and views in the management of the Co-operation, have resulted in the present crisis. Had the members of the committee acted up to their responsibilities as trustees for the nurses, as they ought to have done, this most prosperous society would not have been permitted to incur the financial risks which at present have to be faced unless the requirements of the nurses had been fully met by the Home, nor would an attempt have been made to administer its affairs on any other lines than those which entirely meet the just wishes and requirements of the majority of the nurse members. The interests of the nurse members of the Nurses' Co-operation and of nursing generally render it essential that radical changes shall be made in the present committee so as to secure to the nurses that which belongs to them in its entirety in accordance with the original intentions of those devoted women and others who were the originators of this great organisation of working women.

For the protection of the nurses' interests the Nurses' Co-operation had to be incorporated at the start, and the only form of incorporation available had the disad vantage of being subject to the approval of the Counsel of the Board of Trade and to the limitations imposed upon undertakings incorporated without the word "limited." The time having come, as it has undoubtedly come, when the nurse members of the Co-operation should take the whole management of the Co-operation into their own hands, it will be for them to determine whether they will continue the existing constitution or vote for its modification with a view to their being placed in a position to exercise control over the affairs of the institution and to become full members thereof. There is much to be said, now that the Co-operation is self-supporting and successful, in favour of the nurse members at once taking the management of their own affairs entirely into their own hands. Against this course there arises a difficulty, which the present constitution was a tentative attempt to overcome, due to the fact that the nurse members as working women are constantly away, and cannot attend the necessary meetings regularly or look after the business of management. This difficulty should be capable of solution, and we have no doubt that a conference between the legal gentlemen who so kindly helped the nurses at the start, and have continued to help them throughout, and the nurses would speedily result in a wise solution satisfactory to everybody.

### Appointments.

ALTRINCHAM GENERAL HOSPITAL.—Miss Millicent L. Parker has been appointed night superintendent, and Miss P. Smith sister-in-charge of the theatre and female wards. Miss Parker was trained at the Leamington and Warneford Hospital, and has been two years staff nurse and nearly three years ward sister at the Brentford Union Infirmary, Isleworth. She holds the certificate of the Incorporated Society of Trained Masseuses. Miss Smith was trained at the Brentford Union Infirmary, Isleworth.

BARRY TOWN SURGICAL HOSPITAL.—Miss Annie G. Aldred has been appointed lady superintendent. She was trained at Hammersmith Cottage Hospital and Derbyshire Royal Infirmary, and has since been private nurse at St. George's Home, Sheffield, sister of the male accident ward at Rotherham Hospital, and matron of the Montagu Cottage Hospital, Mexboro'.

FALMOUTH HOSPITAL.—Miss Alice K. Gough has been appointed nurse-matron. She was trained at Bristol Royal Infirmary, and has since been matron at Hampton Court Cottage Hospital, Freshford Cottage Hospital, and Dawlish Infirmary.

HOVE ISOLATION HOSPITAL.—Miss Lilian Baker has been appointed senior nurse. She was trained at Leeds Union Infirmary, and has since been sister at the Royal Chest Hospital, City Road. She has also done fever work at Cambridge and at the Western Hospital, Fulham.

ROYAL HANTS COUNTY HOSPITAL, WINCHESTER.—Miss Barbara Powne has been appointed night sister, and Miss Elizabeth Agnes Marlow sister of the women's ward. Miss Powne was trained at Guy's Hospital, and has since been sister at the General Infirmary, Northampton, Swanley Convalescent Home, and the Royal Victoria Hospital, Belfast. Miss Marlow was trained at the Royal Hants County Hospital, Winchester, and has since been nurse at the Victoria Children's Home, Broadstairs, and the Fountain Fever Hospital, Tooting.

### Maternity Charity and District Nurses' Home.

ANNUAL MEETING.

THE annual meeting of the Plaistow Maternity Charity was held at Grosvenor House on Tuesday afternoon by permission of the Duke of Westminster. The Rev. Canon Hensley Henson presided. It was difficult, he said, to secure a large attendance at this time of year, and on such a fine afternoon, but he hoped that through the Press the effects of the meeting would be wide-reaching. He could speak from personal experience of "London over the Border," having lived for twelve years in Barking. In the recent census results it was shown that the county of Essex stood at the head as far as increase of population was concerned, and it was very striking to notice how soon market gardens and fields were changed into rows of houses and streets. This vast population represented a mass of needs. The people who lived in Barking, East and West Ham, Little Ilford, &c., were not only poor, but they were exposed beyond their fellows to risks of disease and accident. They were living on a reclaimed swamp, and were therefore exposed to various types of illness. Their claim on the inhabitants of the West End was similar to that of the tenants on a country estate: they produced various commodities for the use and luxury of the rich. The gas made at Becton was used by Londoners, and there should be a sense of reciprocal duty. The large sum of £1,000 had been contributed by the district itself towards the funds, and no work had clearer claims on the help of the rich. He proposed a resolution in which the supporters of the charity hailed with hope and gratitude the completion of the home in Howard's Road, and appealed for help to carry on the benevolent work.

This was seconded by Dr. Deacon, who said the Plaistow nurses had been able to reduce the rate of mortality of the district in which they worked. As far as science had gone, it was impossible to entirely prevent the occurrence of puerperal fever, though in a perfect condition of things there should be none. In some districts the deaths from this disease were at the rate of four in a thousand, or even more, since it was at all times difficult to arrive at the true figures; for as the disease was known to be preventable, other causes were assigned. In the present state of knowledge the rate could not be reduced to less than two in a thousand, and this was the rate maintained in the district where the Plaistow nurses worked. He could speak with considerable personal knowledge of the training of the midwives at Plaistow. At the London Obstetrical Society's examinations some of the best midwives were from the training home in Howard's Road.

Mr. R. M. Rowe having given some particulars of the buildings in Howard's Road, Mr. Evans proposed the re-election of the honorary medical officers. This was seconded by Mrs. Wilson.

A vote of thanks to the Duke of Westminster for his kindness in allowing the meeting to be held at Grosvenor House was proposed by the Countess of Selborne, and being duly seconded was carried unanimously.

### Wants and Workers.

Will any kind friend give a district nurse (who works in several parishes) an elastic stocking for a very poor woman who has too large a family to spare any money to purchase one, although she suffers greatly with varicose veins. District Nurse, Haverland, Norwich.

### Echoes from the Outside World.

### AN OPEN LETTER TO A HOSPITAL NURSE.

The Military Exhibition at the Crystal Palace in aid of the Soldiers' and Sailors' Families' Association and kindred societies is rapidly approaching completion, and promises to be a great success. Earl Roberts is going to open it on the 23rd instant, and Lady Roberts will then unveil the great equestrian statue of her distinguished husband, the work of the late Mr. Harry Bates. The work derives additional interest from the fact that it is the only equestrian statue to Lord Roberts at present erected in this country, and those who have ever seen our great general on horseback will admit that he sits a horse as if to the manner born, and thus mounted looks every inch a commander. Many who saw him on the memorable February 2nd, as he rode up and down the streets will endorse this idea from personal experience. At the base of the statue there are two figures of heroic proportions representing War and Victory. The exhibition is to be of very wide scope, embracing every kind of exhibit which illustrates the history and present development of naval and military science. The loan collection of pictures, and relics, and autographs will be especially interesting, and King Edward has promised to lend the Jubilee offerings given to Queen Victoria from the Royal Navy and Marines. The Society world of London is also working heart and soul to make the Exhibition as attractive as possible, in fact, worthy of the charity which is so much thought of by Queen Alexandra, and to which she is so much attached. Amongst those who have given, or are giving, assistance are the directors of the Crystal Palace, who have contributed a magnificent donation of 50,000 guinea season tickets as a free gift to be sold for the benefit of the Society in whose aid the exhibition is held. Mrs. George Alexander has formed a strong sub-committee amongst professional helpers for selling tickets, which includes Miss Fay Davis, Mrs. Beerbohm Tree, Mrs. Esmond, Miss Ellis Jeffreys, Miss Nina Boucicault, Miss Julie Opp, Miss Lena Ashwell, Miss Bergh Faber, Miss Vane Featherstone, and Mrs. Gordon Lennox. The sufferers by the war naturally appeal to all classes of men and women, and it is hoped and believed the Naval and Military Exhibition will score such a triumph that the funds at the disposal of the S. S. F. A. may almost, if not quite, suffice for all the sad cases which deserve the help of a nation grateful for the heroism and the courage of those who fight her battles by sea or land.

---

As the time will undoubtedly come when probationers will take their recreation and district nurses go their rounds on motor bicycles, the progress which vehicles driven by electricity are making in public favour is rather an interesting subject. Those who visited the recent Automobile Exhibition at the Agricultural Hall, London, were of course particularly anxious to see the "Victoriette" which belongs to Queen Alexandra, and in which she can drive herself. It has seats for two, and will travel twenty miles an hour with neither noise nor vibration. It is upholstered in dark morocco leather, has a red carpet, and the fittings and lamps are silver-plated. The King has a couple of automobile carriages for his use at Sandringham, but he does not drive himself, preferring to trust to a machinist. Two countesses have lately ordered electric broughams for themselves, with a box seat for the coachman and footman as in an ordinary carriage, and the idea amongst builders is that these sort of vehicles will be much in demand, especially for evening use, as more damage is done then to valuable horses than at any other time, through waiting about in the cold. It is not only in England that motor cars are coming into favour. Baron Theodore Liebig, who is to be married next month, is going to take his bride for the honeymoon in a large motor car, through Italy, France, and Switzerland, and back to Germany by way of the Tyrol and Vienna. He will drive himself and take no assistant with him. In Rome the Post Office authorities are using motors for the carriage of letters, to convey the postmen to distant points, and to expedite matters. There are also a good many private vehicles driven by electricity, but owing to the extreme narrowness of the streets the number of accidents is rather large, though, apparently, this fact does not influence those who wish to try the novelty of rushing about in a steedless carriage. The sensation is not, however, appreciated by everyone. Some people, after a drive, complain of a sick headache, and others who are bad sailors dislike the resemblance to the thud of the engines on a steamer produced by the electric machinery in a car.

---

Last week I touched upon those pictures in the Academy which bore reference to Queen Victoria. Next in interest come the many canvases of Mr. John Sargent, because they are so much discussed. The most noteworthy is the representation of the two "Daughters of A. Werthheimer, Esq.," whose father was painted by the same artist a year or two ago. The present picture represents two very tall figures in fashionable evening dress, standing close together, one clad in red velvet, the other in shimmering white satin. The taller sister winds her arm around the waist of the shorter, and both of the figures seem instinct with life and vigour. The drapery and the background are very beautiful, but somehow there is a suggestion of coarseness about the whole, which to my mind detracts greatly from its charm. Very different in style is "Mrs. Charles Russell" on the opposite wall, which has a rare merit. The face is a sad and a weary one, but it is painted softly and delicately and the harmony of the black and white, the pale pink and the silver shades is very beautiful. A candelabra which stands on the table at the side appears in some subtle way to throw its subdued light all over the picture. There are two other pictures of Mr. Sargent's which should also be noticed by visitors to Burlington House. One is "Lady Cazalet and her Children," a symphony in crimson, black, and white, and the other "Sir Charles Sitwell, Lady Sitwell, and Family." Here the canvas, though a small one, is sufficiently large to include three children, as well as the parents, and that without any suggestion of crowding. The portraits of the father and mother are excellent, but the children's faces suffer much from want of finish.

---

A portrait which charmed me is "Lady Hickman,' by Arthur S. Cope. The colouring is exquisite, and the whole picture is a delight to the eye. "Herring-Fishers off Kildonan Castle, Isle of Arran," by Colin Hunter, makes one long to flee away and see the scene depicted with its rippling water catching the sunshine and the deep blue grey of the island in the distance. It is a large canvas, and to be thoroughly appreciated should be seen from the other side of the room; but then, alas, the visitors are not transparent, so it is a case of a close view or no view. There are some war pictures: one, "Lindley, Whit Sunday, 1900," by R. Caton Woodville, represents the administration of the Sacrament at morning service, and the effect of the khaki men, the khaki-coloured hills, and the clergyman in surplice and khaki helmet is very strange. Another, called "South Africa, 1901: the Dawn of Peace," by Robert Hillingford, is difficult to understand. The scene is a glen or ravine; Earl Roberts on horseback is reading a proclamation of some sort to his men and others, a nurse in uniform has apparently just left the side of a sick man—to whose lips an Indian bearer is holding a drink—to chase away a bird of evil omen which is flying out of the picture. Lastly, that wonderful veteran, Mr. Sidney Cooper, has four pictures, all of which are as carefully and elaborately painted as if he were twenty-nine instead of ninety years of age.

### View Day at "Barts."

THE second Wednesday in May is the usual occasion for the annual View Day at St. Bartholomew's Hospital, when every portion of the hospital and its accessory buildings are thrown open to the friends and acquaintances of the medical and nursing staff. In the small hours of the morning sisters and nurses, notwithstanding their limited daily allowance of rest, were up and away to Covent Garden, returning to their 6.30 A.M. breakfast with armfuls of flowers and greenery wherewith to decorate the wards. The sisters vie with each other in this self-imposed task, and the results of their artistic efforts and generous expenditure are well repaid by the admiration which is deservedly excited by the floral decorations. In one ward the lavatory basins at the end, for the doctors' use, commonly called "The Slab," was transformed into a kind of reredos by the simple use of branches of wild cherry blossoms and the young beech, just now in its first tender green. Fountains of the snowy flower rose from the basins, while taller sprays waved gracefully over them in all directions. In many instances the predominating colour chosen in the floral decorations was carried out in the hearthrugs and quaint vases on the mantelshelves. Tea was served with lavish hospitality in the sisters' rooms and kitchens off the wards. The patients looked exasperatingly well in their bright jackets and hair tied with ribbons to match the occasion, and all signs of work or anything unpleasant were banished as by the wand of a magician. The nurses wandered where they listed with their friends after the formal business of the day was over. This commences at 2 o'clock sharp, when the treasurer, the committee, and the matron in solemn procession visit each ward in turn. Here they are met by the head surgeons or physicians of the ward, the house-surgeons, the sister, and staff nurses. A report is read by the physician or surgeon of the cases under his care, their condition and the probable length of their stay in the hospital. The matron and sister are then asked if they have any complaints to make, and a similar compliment is in due course paid to one or two representative patients, who one declare themselves to be quite satisfied with existing arrangements. The round of the twenty-eight wards is not completed till after four o'clock, but the merry-making and tea-drinking continue till about 6 P.M., after which the hospital gradually settles down into its normal routine of busy work.

### Novelties for Nurses.

BY OUR SHOPPING CORRESPONDENT.

### UNSHRINKABLE WOOLLEN GARMENTS.

MANY nurses will be glad to know of woollen under-wear which does not shrink. A tendency to shrink is the one objection to the use of wool in garments which have to go constantly to the laundry. The manufacturers of the Griffin brand of under-wear have overcome this objection, and at the same time have produced a material for the purpose of the highest class, and the garments made of it are excellently shaped and finished. The manufacturers are very practical, and they have made a speciality of hose, which are woven with double feet, rendering the "life" of socks and stockings twice as long as usual. Durability and comfort are the special characteristics of the Griffin brand under-wear—which any draper will procure—and it should especially appeal to nurses, whose clothing has very often to stand a strain at the hands of the careless laundress, the nurse being seldom in a position to select her own.

### THE "LITO" FLOUR.

THIS is a flour which should recommend itself to all desiring a commodity of good quality, and one that saves time. It is a self-raising flour—that is it has mixed with it ingredients of the nature of what is known as "baking powder," and which are of a harmless and tasteless character. They are judiciously mingled with the flour, so that anyone wishing to make cakes, scones, or the like, will find their task greatly simplified. The "Lito" Flour is a speciality of the "Hovis" Bread Company, who very well know how to suit the public taste.

### THE BEST EAU DE COLOGNE.

THE summer is coming on, and with it comes the difficulty of keeping the invalid cool and refreshed. Eau de Cologne is an immense help in the circumstances. It is always welcome, when scents of a different character are objected to. But the quality must be good. Therefore, do not hesitate to recommend the well-known 4711 Eau de Cologne, which is sold everywhere, and at the chief depôt, 62 New Bond Street. All sorts of scents are sold under the name of Eau de Cologne, all purporting to come from that city. Many are very inferior; I know of none to surpass the 4711 Eau de Cologne in excellence.

### Everybody's Opinion.

[Correspondence on all subjects is invited, but we cannot in any way be responsible for the opinions expressed by our correspondents. No communication can be entertained if the name and address of the correspondent is not given, as a guarantee of good faith but not necessarily for publication, or unless one side of the paper only is written on.]

### THE NURSES OF BIRKENHEAD HOSPITAL.

"OXTON" writes: On enquiry the matron of the Borough Hospital, Birkenhead, tells me that the statement about the nurses' sleeping in "cellars" was incorrect. The servants sleep in the basement—it can hardly be called cellar. The new accommodation is badly required for increase of staff, and also because the present bedrooms are too near the wards.

### "THE GUILTY FEW."

"A SOMERSETSHIRE NURSE" writes: May I give a hint to the "guilty few" who advertise their vacancies in your columns that when a stamp is enclosed for a reply they should certainly use it, if only to say "filled" or "unsuitable." It is trying to those who have not pennies to waste that the cost of some advertisement is probably more than covered at their expense—if the applications are as numerous as is said to be the case—and all the stamps are kept.

### THE NURSES' CO-OPERATION.

"A 'CO.' NURSE" writes: I think your article of May 11th in THE HOSPITAL "NURSING MIRROR" requires a little correction. The Nurses' Co-operation was founded in 1891 by nurses for nurses. I have before me the first annual report of the Co-operation, from which I quote the following:—"The plan of the Co-operation first originated with Miss Belcher, one of the first nurses, and it started with a capital of £500, £300 being advanced by two gentlemen on the committee and £200 by four of the nurses."

"MISS HELEN KEENE" writes: In your article in THE HOSPITAL of May 11th you say the Co-operation was founded by Mr. Capel Slaughter, Sir Henry Burdett, Mr. Charles Cheston, and the late Mr. Walter Burns, and that these gentlemen gave ungrudgingly of their time and brains to secure for nurses a safe and adequate return for their services. As one of the first members of the Co-operation I always thought that it was founded by the nurses themselves, and on turning to one of the first reports I find it stated: "These nurses, feeling the need of a central office, wrote to THE HOSPITAL last year, and two of them, Miss Napper and Miss Belcher, came forward and formulated a scheme," and further on in the same report: "Four nurses have advanced their savings to help with the preliminary expenses, and two members of the committee have added to the sum thus made available for the start." These two gentlemen were Mr. Charles Cheston and Mr. Capel Slaughter. The four nurses were Miss Belcher, Miss Napper, Miss Ward, and Miss Brant. Neither Sir Henry Burdett nor the late Mr. Walter Burns was named in the first annual report as a member of the executive committee.

[We have dealt with the points raised by our correspondents in the article giving the history of the Nurses' Co-operation, which appears on page 97. Our correspondent, Miss Keene, will find, if she refers to the first report, that the names of both Mr. Capel Slaughter and Sir Henry Burdett appear therein as members of the committee. Mr. Walter Burns' part in the early days of the Co-operation is fully set forth in the article on page 97.—ED. THE HOSPITAL.]

### CROYDON UNION INFIRMARY.

"MATRON" writes: I think it is a pity that the Croydon Board of Guardians cannot be left to manage their own affairs—which I am sure they are capable of doing—without the constant interference of people who know absolutely nothing about the matter, and much less about the duties of a matron. If I may be allowed to speak very plainly, it is nurses who require weeding out, not matrons. "A Woman of No Importance" little knows the anxious hours that matrons pass trying to improve some nurses, who in the end prove an utter failure. They will neither be led nor governed.

"FLORENCE MEYRICK," Nursing Hostel, 33 Beaumont Street, London, writes: From her own words "A Woman of No Importance" shows that a matron who does her duty in refusing a certificate to an inefficient nurse is not right in the eyes of that nurse, whether she refuses the certificate at the end of three years, or, as her matron did, dismiss her at the end of six months. If a nurse wishes for a good certificate, she should work for it, as everyone else has to work for certificates, and not go on in a slovenly, unsatisfactory way during her training, and then expect a good certificate at the end of it. A certificate would be of no value if it were given to good and bad alike. Depend upon it, a good nurse will get a good certificate. I have two or three times taken a nurse, thinking that she had been, perhaps, a little hardly used, but I have never done so without, before long, coming to the conclusion that her former matron was right in her judgment of her. Many nurses nowadays seem to think that they exist for the benefit of the nursing profession; but I submit that they exist for the benefit of the sick, and that it is a cruel wrong to give a certificate of nursing proficiency to an unsatisfactory woman, either in work or conduct. If this view were more thoroughly taken to heart by nurses, we should hear fewer complaints of their treatment and more eulogies of them from their patients.

### A DEVOTED MATRON.

"A NURSE IN SOUTH AFRICA" writes: The death of Miss Ellen Kayser, Matron of the Plague Camp at Capetown, is a source of deep regret and grief to all who knew her. The deceased lady was the daughter of the Rev. Fred Kayser, of the Knappshote Mission Station. There she learnt Dutch and Kaffir; subsequently she continued her studies at Stellenbosch, and eventually found her way to Kimberley, where she trained as a hospital nurse. Before the war broke out, Miss Kayser was on the staff of the Johannesburg Hospital, but came down to Capetown in September and took up private nursing. Two months ago, she was nursing a patient at Rondesbosch, who was found to be suffering from plague; this was the first case which had occurred at that time. Miss Kayser then offered her services to the Government, and she and another nurse were the first to take duty at the Plague Hospital, where she worked nobly until her death.

### NURSES IN THE MISSION FIELD.

"DR. HERBERT LANKESTER," of the medical department of the Church Missionary Society, Salisbury Square, writes: Some weeks ago you published an account of a meeting held in connection with our Medical Mission Auxiliary. As a result of this article a considerable number of nurses have written to us asking if we could give them work in our mission hospitals abroad. I think it might be well if you would kindly make it known that the C.M.S. sends out no women simply as nurses. They go out distinctly as missionaries, only using their nursing knowledge in order to get into touch with the people among whom they work. In the same way we do not send out medical men or women simply with the object of their doing medical work, while clergymen or others do the spiritual work; but they go out distinctly as missionaries, being responsible both for the preaching of the Gospel and the healing of the sick. I might also say that at present we are needing fully-trained nurses in different parts of the world, but they must also feel called to go out as missionaries. We should always be pleased to hear from such.

### NUNS AS NURSES.

"MAY E. MOORE" writes from Desert Serges Rectory, Eniskean, County Cork: Having read with interest the correspondence concerning nuns as nurses, may I as one who has had considerable experience in the matter give my opinion? There are undoubtedly capable nurses, women devoted to their duty in their ranks, but in some of the so-called nursing orders there are many going out to tend the sick who, prior to their first appearance as private nurses, have never seen the inside of a hospital. I was for some years matron of a foreign hospital, where, through stress of work, nuns were often summoned as extra nurses. One young English nun arrived one evening to take charge of all patients, several among whom were seriously ill with typhoid. On my inquiring in what hospital she had been trained, she replied: "I have never been inside a hospital before." It appears that some of the older sisters, who used to be trained in a French hospital, instruct the new comers in the convent in the use of the thermometer, &c., but leave them hopelessly ignorant of bed-making, dressing wounds, making poultices, or the art of persuading unwilling patients to take nourishment. Their most important duty seems to consist in saying a certain number of prayers, and to find time for these even dying patients must be neglected. One night the electric wires coming in contact, the sister on duty woke each patient in turn to inquire if he or she had rung, instead of consulting the index with the number of each room, and finally came to me with the same query just as I was coming out to stop the continuous ringing. Having complained to the Mother Superior she sent us some seven or eight of her staff in turn, but by putting on cold wet poultices, scalding a patient's feet severely with a hot bottle, leaving patients critically ill without nourishment, showing incapacity to dress wounds, and, as a matter of course, leaving soiled and saturated sheets unchanged during the night, they made it impossible for us to continue employing them. They also persistently refused to wash up any cups, glasses, &c., used by them during the night, much to the indignation of our own staff. Apart from their work one could not help liking the nuns for their pleasant, courteous manners, but their idea of nursing seemed to be the amateur one, that it consists in shaking up pillows and giving drinks, if the patient wishes to take them.

### For Reading to the Sick.

ASCENSIONTIDE.

HAIL! festal day! to endless ages known,
When God ascended to His starry throne.

Now with the Lord of new and heavenly birth
His gifts return to grace the springing earth.

Now Christ from gloomy hell comes triumphing;
And field and grave with flowers and leafage spring.

The reign of hell o'erthrown, He mounts on high,
Sent forth with joyous praise from sea and sky.

*Powell.*

"A CLOUD RECEIVETH HIM OUT OF THEIR SIGHT."

When Christ went up to Heaven the Apostle stayed
Gazing at Heaven with souls and wills on fire,
Their hearts on flight along the track He made,
Winged by desire.

Their silence spake: "Lord, why not follow Thee?
Home is not home without Thy Blessed Face,
Life is not life. Remember, Lord, and see,
Look back, embrace.

"Earth is one desert waste of banishment,
Life is one long-drawn anguish of decay.
Where Thou wert wont to go we also went:
Why not to-day?"

Nevertheless a cloud cut off their gaze:
They tarry to build up Jerusalem,
Watching for Him, while thro' the appointed days
He watches them.

They do His Will, and doing it rejoice,
Patiently glad to spend and to be spent:
Still He speaks to them, still they hear His Voice
And are content.

For as a cloud received Him from their sight,
So with a cloud will He return ere long:
Therefore they stand on guard by day, by night,
Strenuous and strong. *C. Rossetti.*

Are we ascended with Christ in heart and mind, dwelling in heavenly places with Christ? Are we living apart from the world? Often conquered by it, perhaps often falling, often feeling our negligencies and ignorances of a thousand kinds, but still, in the eye of God, living separate from the world? What practical difference have the Resurrection and Ascension of our Lord made in the lives of thousands of baptised persons? Ay! or in the lives of hundreds who are communicants?—*Bishop Wilkinson.*

Even in His triumphal Ascension, the Lord forgot not that He was a Saviour, and that He had finished the work He came on earth to do. It was the Good Shepherd who went up into Heaven and called upon the Heavenly Choirs to rejoice with Him over us whom He had redeemed. . . . Blessed be our ascended King, our Chief Shepherd who is gone into Heaven and is on the right hand of God; angels and authorities and powers being made subject unto Him. Alleluia!—*From "Devotional Helps."*

We ought, most beloved brethren, thither to ascend in heart, where we believe Christ to have in body ascended. Let us flee earthly desires; let us, who have a Father in Heaven, take henceforth no delight in things below. And this is exceedingly to be considered by us, that He who hath ascended in meekness will return in terror; and whatsoever He hath with gentleness enjoined us, He will exact with severity.—*S. Gregory.*

### Notes and Queries.

**The Editor is always willing to answer in this column, without any fee, all reasonable questions, as soon as possible.**
**But the following rules must be carefully observed:—**
**1. Every communication must be accompanied by the name and address of the writer.**
**2. The question must always bear upon nursing, directly or indirectly.**
**If an answer is required by letter a fee of half-a-crown must be enclosed with the note containing the inquiry.**

*Crippled Children.*

(65) Kindly tell me how I can obtain the care of crippled children as described by Mrs. Humphrey Ward in THE MIRROR of January 26th.—*Esther C.*

Apply the School for Crippled Children, Passmore Edwards Settlement, Tavistock Square.

*Shade.*

(66) Kindly tell me where I can get shades to clip on candle, to shield the light from patient's face.—*Reg.*

At the lamp department of any good shop.

*Nurses' Abstinence League.*

(67) Will you kindly give me the address of the Nurses' Abstinence League?—*Sister Gertrude.*

The Hon. Secretary, the Nurses' National Total Abstinence League, 47 Oakley Street, Chelsea, S.W.

*Rome.*

(68) Will you tell me the address of the Protestant Nursing Institute opened in Rome? Do you think it a good opening for fully-trained English nurses wishing to go abroad?—*Nurse McG.*

It is exceedingly difficult to obtain reliable information respecting private institutions abroad. You should write to the English Consul for information before incurring any expense.

*Military.*

(69) Is there any institution for destitute and mentally afflicted daughters of military men? The poor lady in question, though harmless, has no relatives in a position to help her, and has only a pension of £24 yearly.—*M. L. A.*

The Secretary, the Soldiers and Sailors' Families Association, 23 Queen Anne's Gate, Westminster, S.W., or the Royal Military Benevolent Fund, 193 Queen's Gate, S.W., would be able to advise you as to this case.

*St. Barnabas.*

(70) Will you kindly give me some particulars of the Guild of St. Barnabas for Nurses?—*E. K.*

The Guild of St. Barnabas is intended to help nurses who are members of the English Church in their professional and religious life. Address the Secretary, the Nurses' Hostel, Francis Street, W.C.

*Better Practice.*

(71) Can you suggest how I can get better practice? I am a maternity nurse, and whilst at times I might have two cases at the same time, at others I have none.—*Nurse O'D.*

This is the common experience of all nurses. Your connection will increase as you become better known to the medical men, that is, of course, if you show yourself competent and pleasant.

*Cards.*

(72) Would you kindly tell me what particulars a nurse should have printed on her professional cards; and how should they be set out?—*X. Y. Z.*

The particulars should be as concise as possible as to training, experience and fees. Consult a good stationer as to the arrangement: it should be neat, clear, and pretty.

*Magnetism.*

(73) I should be pleased to have your opinion respecting magnetism in cases of nervous debility. My sister, who is a public worker, has for some months been suffering from what appears to be nervous exhaustion. Her nerves were so bad last autumn that she could not walk out alone. She was under medical treatment for some time, and that, with maltine, good food, and fresh air, in some degree restored her to her usual health. With the spring, however, there was a recurrence of the old symptoms, but with less shakiness in the limbs generally. We have been advised to get a set of magnetic appliances costing in all five guineas. It seems rather a large sum of money to spend if it is likely to prove useless. I should feel much obliged if you would tell me if you approve of magnetism in nerve cases, and if you know the firm whose name I enclose.—*Nurse C.*

The use of magnetism in medicine is still quite in the experimental stage. It may be accepted as a general principle that in those cases in which either magnetism or electricity is of service its application requires to be as carefully adjusted to the case as is the case with drugs or any other method of treatment. Hence even if the advertised magnetic appliances possessed any efficacy, which in most cases may be doubted, it would be entirely inadvisable to make use of them for the treatment of a case except under qualified medical advice.

**Standard Books of Reference.**

**"The Nursing Profession: How and Where to Train." 2s. net: post free 2s. 4d.**
"Burdett's Official Nursing Directory." 3s. net.; post free, 3s. 4d.
"Burdett's Hospitals and Charities." 5s.
**"The Nurses' Dictionary of Medical Terms." 2s.**
**"Burdett's Series of Nursing Text-Books." 1s. each.**
**"A Handbook for Nurses." (Illustrated.) 5s.**
**"Nursing: Its Theory and Practice." New Edition. 3s. 6d.**
**"Helps in Sickness and to Health." Fifteenth Thousand. 5s.**
**"The Physiological Feeding of Infants." 1s.**
**"The Physiological Nursery Chart." 1s.; post free, 1s. 3d.**
**"Hospital Expenditure: The Commissariat." 2s. 6d.**
**All these are published by THE SCIENTIFIC PRESS, Ltd., and may be obtained through any bookseller or direct from the publishers, 28 and 29 Southampton Street, London, W.C.**

